# An Efficient Sampling-Based Algorithms Using Active Learning and Manifold Learning for Multiple Unmanned Aerial Vehicle Task Allocation under Uncertainty

**DOI:** 10.3390/s18082645

**Published:** 2018-08-12

**Authors:** Xiaowei Fu, Hui Wang, Bin Li, Xiaoguang Gao

**Affiliations:** 1 School of Electronics and Information, Northwestern Polytechnical University, Xi’an 710129, China; huiw@mail.nwpu.edu.cn (H.W.); cxg2012@nwpu.edu.cn (X.G.); 2 Shaanxi Key Laboratory of Integrated and Intelligent Navigation, Xi’an 710068, China; emlibin@163.com

**Keywords:** uncertainty, multi-UAVs, task allocation, active learning, manifold learning

## Abstract

This paper presents a sampling-based approximation for multiple unmanned aerial vehicle (UAV) task allocation under uncertainty. Our goal is to reduce the amount of calculations and improve the accuracy of the algorithm. For this purpose, Gaussian process regression models are constructed from an uncertainty parameter and task reward sample set, and this training set is iteratively refined by active learning and manifold learning. Firstly, a manifold learning method is used to screen samples, and a sparse graph is constructed to represent the distribution of all samples through a small number of samples. Then, multi-points sampling is introduced into the active learning method to obtain the training set from the sparse graph quickly and efficiently. This proposed hybrid sampling strategy could select a limited number of representative samples to construct the training set. Simulation analyses demonstrate that our sampling-based algorithm can effectively get a high-precision evaluation model of the impact of uncertain parameters on task reward.

## 1. Introduction

Recently, multiple unmanned aerial vehicles (UAVs) have received increasing attention for their accomplishments in both military and civil applications [1,2,3,4]. Task allocation is the critical basis of multi-UAV collaborative control, and it is to determine which UAV within a multi-UAV fleet should perform which task, in order to achieve the desired task execution effect with the maximum task efficiency at the lowest cost [5]. Since the actual environment is often complex, dynamic and full of uncertainties, the task allocation of many UAVs often needs to be carried out in an uncertain environment. In order to enhance the robustness of the task assignment algorithm, multi-UAV task assignment methods in uncertain environments have become a hot topic [6,7,8].

There is some research on multi-UAV task allocation problems under uncertainty. A lot of task allocation problem models [5,9] and task assignment solving algorithms [10,11,12,13,14,15] have been developed to meet the respective needs of various situations. Some intelligent methods have been proposed for multi-UAV task allocation problem under uncertain situation [7,8,16,17,18,19]. References [16,17,18] used the concepts of interval uncertainty to model the uncertain factors of task allocation problem and the traditional auction algorithm, genetic algorithm and particle swarm optimization (PSO) are separately used to solve multi-UAV task allocation problems under uncertainty. Ponda [8] proposed that the uncertainties in the true environment can be captured as parametric uncertainties in the underlying system models, which can affect various portions of the planning model. Robust planning algorithms were developed to select the best task assignment in order to minimize the effect of uncertainties on the final task score. For parametric uncertainties, Ponda [8] and Whitbrook [19] used robust strategies to capture the propagation law of uncertainties in the score function and calculates the expected reward scores to participate in the task allocation process.

The main difficulty is how to analytically compute these robust scores [8]. The underlying mapping from uncertain parameters to score is usually unknown and can be highly nonlinear. In those cases, approximate scores can be more easily computed using sampling-based procedures with the Monte-Carlo method, and the weighted score can be obtained from various realizations of the uncertain parameters, but using Monte Carlo sampling methods requires too many samples [20]. 

Machine learning has also been conducted in the field of multi-UAVs collaborative control. Machine learning approaches offer the potential to more efficiently sample the uncertainty space with fewer number of samples, but simultaneously minimize the impact on the accuracy of the robust score metrics associated with fewer samples [21,22,23,24,25,26,27]. Quindlen [20] used Gaussian process regression (GPR) and machine learning active learning algorithms (AL) to replace Monte-Carlo-based evaluation methods, and this effectively reduces the computational time complexity and sampling costs. However, the iterative training selection process of active learning requires a large amount of calculation, so we need to further improve the sample selection mechanism. 

Tang [28] expressed the concept of sample representation and uncertainty, and considered the two factors to evaluate the amount of information carried by the sample, and used this to select the samples needed for the training process, and achieved smaller Root Mean Square Error (RMSE) than only considering a single factor. Zhou [29,30] used the manifold learning algorithm to preliminary select the samples from unlabeled sample pools and evaluate them, which reduced the classification error rate effectively. These approaches for classification problem can be combined with existing active learning in regression problem for the score prediction of sample.

This paper focuses on the task assignment of multi-UAVs with time-window constrain, which considers the uncertainty of task duration, and evaluates the impact of uncertain parameters on the task’s reward. Based on the existing active learning sampling algorithm, we adopt manifold learning to design multi-points simultaneous sampling strategies. The main contributions of this paper contain two aspects as follows:

- Multi-points simultaneous sampling is introduced into active learning to obtain the training set quickly and efficiently. That is, multiple samples are selected before retraining the regression model, so that computational costs can be reduced by reducing training steps for same number of samples without decreasing the accuracy.
- We proposed an improved hybrid sampling strategy based on manifold learning and active learning. Only using active learning may lead to sample agglomeration under the framework of multi-points simultaneous sampling. Manifold learning method is used to screen samples in advance, which constructs sparse graph to represent the distribution of all samples through a small number of samples. This strategy could select a limited number of samples that with good representativeness to construct the training set.

The remainder of this paper is organized as follows: Section 2 provides a robust task assignment model and solving method. Section 3 describes the Gaussian process regression and active learning algorithm. Section 4 proposes our improved hybrid sampling algorithm proposed. Section 5 presents computational experiments and interprets the results. Section 6 summarizes the conclusions drawn from our research, and points out the future directions of this work.

## 2. Robust Task Assignment Model and Solving Method

### 2.1. Task Allocation Problem in Uncertain Environment

The multi-UAV task allocation problem is a multi-constraints and multi-coupled combinatorial optimization problem containing timing-constraints. In this paper, we use the CMTAP [9] model to describe this optimization problem. Given a formation consisting of *N_a_* UAVs to perform tasks on *N_t_* targets, and the goal of task assignment is to find a feasible allocation scheme to optimize reward function, expressed as Equation (1):

(1) $$\left\{ \begin{array}{l} \underset{x}{\max}\sum_{i=1}^{N_{a}}\sum_{j=1}^{N_{t}}c_{ij}(x,\theta )\\ s.t.\;G(x,\theta )\leq b\\ x\in \chi \;\chi ={\left\{0,1\right\}}^{N_{a}\times N_{t}} \end{array}\right.$$

where, *c_ij_* is the reward score that UAV *i* get from performing one task on target *j*, θ is an allocation parameter related to score function calculation. G(x,θ)≤b represents the relevant constraints for task assignment, χ is 0–1 decision variable set. 

The main constraint is the time-window constraint. If a task must complete within a specified time range, the task is said to have time-window constraint. In the CMTAP model, the time-window constraints usually exist for dynamic targets or time-sensitive targets. The time-window constraints can described as Equation (2):

(2) $$ET_{j}\leq t_{j}\leq LT_{j}$$

where, $ET_{j}$ and $LT_{j}$ are the earliest time limit and the latest time limit for performing the task *j* respectively, $t_{j}$ is the execution time of the task *j*.

Under the above time window constraints, the objective optimization function can be established as Equation (3):

(3) $$c_{ij}=\left\{ \begin{array}{l} Value_{j}\;\exp(-\lambda (t_{j}-ET_{j}))-\alpha *L(V_{i}T_{j}),ET_{j}\leq t_{j}\leq LT_{j}\\ 0,\;otherwise \end{array}\right.$$

where, on the basis of the time window of task, the optimization goal of the task shortest completion time is taken into consideration. The optimization function is established as the time index discount model [8], which means the task score decreases with the increases of the task execution time in the time-window. $Value_{j}$ is the value of task *j*, $\left[ET_{j},LT_{j}\right]$ is the time-window of task *j*, λ is the parameter of the time index discount model which is used to reduce the nominal value $Value_{j}$ according to the delay $t_{j}-ET_{j}$; α is distance penalty factor which represent the fuel consumption of UAVs, $L(V_{i}T_{j})$ is distance between UAV *i* and target *j*.

This paper studies the problem of task allocation under parameter uncertainty. In specific multi-UAV task allocation problems, uncertain parameters often have their own distribution, which can be obtained from historical data, surveys or theoretical calculations. The conventional distributions have uniform distribution, normal distribution, gamma distribution and so on.

Traditional task assignment assumes that the execution of the task is performed for a short period of time or is completed instantaneous. However, considering the complexity of the real environment, each UAV has its own task duration due to its own characteristics, load capacity, and movement factors, and this execution duration is still uncertain and is a random process. And uncertain execution duration may lead to a particular task allocation result become completely infeasible in realistic environment. 

To simplify the complexity of the problem, we focus on the strategy to deal with uncertain parameters and just consider task duration is uncertain. The strategy could also apply to other scenario with other uncertain parameter or many uncertain parameters at the same time. While the true value of *θ* is unknown, it is assumed that a likelihood model of the uncertainty parameter is known beforehand. The task duration $t_{jduration}$ obeys a statistical probability distribution, which is known as Equation (4):

(4) θ~P(θ)

### 2.2. Task Allocation Method Based on CBBA under Parameter Uncertainty

Ponda [8] established a robust model of the expected value method and solved it based on the CBBA algorithm. The CBBA consists of two phases separated from each other: the task bundle construction phase and the conflict resolution phase. The algorithm loops and iterates in the task bundle construction phase and the conflict resolution phase until all the agent tasks are assigned in a consistent manner. 

The uncertainty of the task duration $t_{jduration}$ will affect the reward score of each task in UAV’s task bundle. Therefore, in order to enhance the robustness of the task assignment algorithm, it is necessary to deal with the uncertain parameters in the assignment process. 

Robust strategies can evaluate the influence of uncertain parameters θ on the task’s reward score, that is, capture the uncertainty of parameters distribution in the score function through this strategy:

(5) $$\underset{x,\tau }{\max}E_{\theta }\{\sum_{i=1}^{N_{a}}\left(\sum_{j=1}^{N_{t}}c_{ij}(x,\theta )x_{ij}\right)\}$$

The robust strategy is to obtain the expected value of the task’s reward score by different values of uncertain parameter, and then to maximize the expected value to obtain an optimized task assignment result.

When an UAV constructs its own task bundle, the task bundle score is calculated as Equation (6):

(6) $$J_{p_{i}}=E_{\theta }\{\sum_{j=1}^{N_{t}}c_{ij}(\tau_{ij}*(p_{i}),\theta )x_{ij}\}=\int_{\theta \in \Theta }(\sum_{j=1}^{N_{t}}c_{ij}(\tau_{ij}*(p_{i}),\theta )x_{ij})P\left(\theta \right)d\theta =\int_{\theta \in \Theta }J_{p_{i}}(\theta )P(\theta )d\theta$$

where, $p_{i}$ is CBBA information structure task execution timing, $\tau_{ij}*$ is the optimal execution time of each task in the task bundle under specific parameter θ.

Based on CBBA, the multi-UAV task allocation problem under uncertainty can be solved. In the task bundle construction phase, each UAV uses the expected score of the task bundle to compute the bid for each bidding task, and selects new tasks for the construction that can maximize the reward score of its task bundle. In the conflict resolution phase, each UAV performs a consistency negotiation through communication, and this will result in a conflict-free optimization task allocation. Since the expected score captures the influence of the uncertain parameter on the task’s reward score, the task allocation results generated will achieve better execution effect under actual situation.

## 3. Gaussian Process Regression and Active Learning Algorithm

It’s difficult to get the relationship between uncertain parameters and task reward scores analytically, so sampling-based approximations are used instead, but the samples are not easy to obtain. In order to evaluate sampling-based approximations effectively, Gaussian process regression models are trained from a small set of samples, which are iteratively selected by active learning. This section presents the machine learning procedure for sampling-based approximations based on Gaussian Process regression and Active Learning (GPAL).

### 3.1. Approximate Expected Reward Calculation Method Based on Gaussian Process Regression Model

The expected task reward score of Equation (6) is difficult to analytically calculate. Ponda [8] used Monte-Carlo sampling method to approximate the expected value of the task’s reward. The disadvantage of using the Monte Carlo sampling method is that there are too many sampling points and the sampling cost is too high. The GPAL algorithm [20] can perform the sampling of uncertain parameters of the robust allocation method, achieving a reduction in the number of samples without degrading the evaluation accuracy.

Gaussian process refers to a set of random variables, and any finite random variable in this set obeys the joint Gaussian distribution [21]. A Gaussian process can be viewed as a distribution over possible functions. The most important application of gaussian process is to solve regression problem [21]. In particular, Gaussian process (GP) regression models return mean and covariance functions that both predict the unknown true function’s response and quantify the confidence in those predictions [20]. 

Given a data set consists of N samples $\theta =\{\theta_{1},\theta_{2},\ldots \theta_{N}\}$, and its corresponding dependent variable is $J_{\theta }=\{J_{\theta_{1}},J_{\theta_{2}},\ldots J_{\theta_{N}}\}$. Their mapping relationship is expressed as Equation (7):

(7) $$J_{\theta }=J(\theta )$$

where, $J_{\theta }$ is mission score, J is the evaluated relationship between θ and $J_{\theta }$.

For the robust task allocation problem mentioned above, this Gaussian Process is specified as Equation (8):

(8) $$J\left(\theta \right)=GP(m(\theta ),k(\theta ,\theta^{\prime }))$$

where, θ and $\theta^{\prime }$ are in either the training or the test sets, m(θ) is the mean function and $k(\theta ,\theta^{\prime })$ is the covariance function (the kernel function of Gaussian Process Regression Model):

(9) $$m(\theta )=E_{\theta }[J(\theta )]$$

(10) $$k(\theta ,\theta^{\prime })=E_{\theta }[(J(\theta )-m(\theta ))(J(\theta^{\prime })-m(\theta^{\prime }))]$$

In this work, m(θ) is preprocessed to *0*, $k(\theta ,\theta^{\prime })$ uses the squared exponential.

(11) $$k(\theta ,\theta^{\prime })=\alpha^{2}\exp(-\frac{1}{2}(\theta -\theta^{\prime })\Lambda^{-1}{\left(\theta -\theta^{\prime }\right)}^{T})$$

where, GP hyperparameters $\left(\alpha^{2},\Lambda \right)$ are selected during the training process; hyperparameters of the GP are generally achieved by maximizing the marginal likelihood function, that is to say, minimize the negative logarithmic marginal probability with respect to hyperparameters using conjugate gradient method:

(12) $$L=-\log p(J_{P_{i}}(\theta )|\theta )$$

Set training set $S:\{\theta_{S},J_{p_{i}}(\theta_{S})\}$, prediction sample point $\theta_{*}\in \Theta$, $\theta_{S}$ and $\theta_{*}$ is in line with the joint prior distribution:

(13) $$\left[\begin{array}{l} J_{p_{i}}(\theta_{S})\\ \hat{J_{p_{i}}(\theta_{*})} \end{array}\right]\sim N(\left[\begin{array}{l} \mu (\theta_{S})\\ \mu (\theta_{*}) \end{array}\right],\left[\begin{array}{l} k(\theta_{S},\theta_{S})\;k(\theta_{S},\theta_{*})\\ k(\theta_{*},\theta_{S})\;k(\theta_{*},\theta_{*}) \end{array}\right])$$

According to the Bayesian regression method, we can find a $\hat{J_{p_{i}}(\theta_{*})}$ posterior distribution, a Gaussian distribution:

(14) $$\hat{J_{p_{i}}(\theta_{*})}\sim N(\mu (\theta_{*}),\sum \left(\theta_{*}\right))$$

where, $\mu (\theta_{*})$ and $\sum \left(\theta_{*}\right)$ are represented as Equations (15) and (16):

(15) $$\mu (\theta_{*})=k(\theta_{*},\theta_{S})K^{-1}J_{p_{i}}(\theta_{S})$$

(16) $$\sum \left(\theta_{*}\right)=k(\theta_{*},\theta_{*})-k(\theta_{*},\theta_{S})K^{-1}k(\theta_{S},\theta_{*})$$

(17) $$K=k(\theta_{S},\theta_{S})$$

The sample set is broken up into two subsets, the sampled subset (the training set) labeled *S* and the not-sampled subset labeled *U* (the remaining samples except *S*). Set *S* contains a small collection of sampled vectors θ and their corresponding true scores. Set *U* is significantly larger and only contains uncertain parameter θ. The GPR model is trained by the training set *S*, and then the GPR model predicts the task reward score of all samples in the entire sample set (As scores in *S* are explicitly known, the training samples in *S* will have zero prediction error with a noise-free GPR).

With known probability distribution of θ, according to the [23,24,25], the corresponding weights are calculated as in Equation (18):

(18) $$\omega_{k}=P(\theta_{k})/\sum_{k=1}^{N}P(\theta_{k})$$

The expectation score is approximately calculated as Equation (19):

(19) $$\hat{J_{p_{i}}}\approx \sum_{k=1}^{N}\omega_{k}\mu (\theta_{k})$$

### 3.2. Sampling Strategy Based on Active Learning

If the training set is not selected effectively, the evaluation error $\hat{J_{p_{i}}}-J_{p_{i}}$ will be large. The training set of the GPR model can also be constructed intelligently in order to improve the accuracy of these predictions. The active learning algorithm selects the best sample iteratively by a specific evaluation strategy. Different from the passive learning algorithm (using all existing samples for the training process), the best training sample set constructed can reduce the evaluation error $\hat{J_{p_{i}}}-J_{p_{i}}$. And the number of samples required to be sampled for training can be reduced without decreasing the evaluation accuracy. 

On the one hand, the reduction on size of the training set can reduce the total sampling costs during the construction of the training set. On the other hand, the training costs of GPR model have also been reduced. The computational complexity of the hyperparameters optimization Equation (12) and the posterior distribution inference Equation (14) for prediction correlates strongly with the dimension of uncertain parameters θ. 

This paper adopts pool-based active learning, which means samples are chosen from an unsampled pool and queried to construct the training set [26]. The crux to the active learning framework is to design evaluation strategies to evaluate possible samples and then select the best samples to training set. We are committed to improving the precision of approximate expected score of Equation (19). The true expected score Equation (6) is an expected model. The best and informative sample is the most uncertain sample [27], which could improve the evaluation accuracy and minimize the entropy of the total integrand $J_{p_{i}}(\theta )P(\theta )$ from Equation (6) [23]. This entropy reduction metric is known as the uncertainty sampling metric [23]. Quindlen [20] modified earlier active learning regression methods for Bayesian quadrature to create an iterative process to select samples and retrain GPR model [22].

Equation (6) suggests that the most appropriate selection criteria is not only a function of $\hat{J_{p_{i}}(\theta )}$, but also the function of distribution probability P(θ). Selected samples should ignore the little-probability regions, which will have little effect upon $\hat{J_{p_{i}}}-J_{p_{i}}$ even if the accuracy was improved in that region. The variance corresponding to integration function can be adopted as the information entropy evaluation criterion of sample [23,27].

According to the above strategy, a sample $\overset{-}{\theta }$ with maximum variance of integration $J_{p_{i}}(\theta )P(\theta )$ should be selected to minimize the entropy of the total integrand. The variance of integration function is Equation (20):

(20) $$V[J_{p_{i}}(\theta )P(\theta )]=\sum \left(\theta \right)P{\left(\theta \right)}^{2}$$

The best sample $\overset{-}{\theta }$ could be selected by the sampling function, formulated as Equation (21):

(21) $$\overset{-}{\theta }=\underset{\theta }{\arg\max}\{V[J_{p_{i}}(\theta )P(\theta )]\}=\underset{\theta }{\arg\max}\{\sum \theta P{\left(\theta \right)}^{2}\}$$

The points selected according to the above strategies can minimize $\hat{J_{p_{i}}}-J_{p_{i}}$ at the same time. Active learning selection strategy is shown as Algorithm 1, which mainly presents the procedure of the selection during each iteration. Each sample θ of not-sampled set *U* should be calculated the evaluation value according to sampling function. Firstly, we use GPR model trained in last iteration to predict posterior variance. Then, we multiply predicted variance with square of corresponding θ probability as the evaluation value. Finally, we select $\overset{-}{\theta }$ with the largest evaluation value to join in the training set.


**Algorithm 1**
1: Input: GRP model, *U*(*m_t_*); Output: $\overset{-}{\theta }$ 2: For each θ in *U* 3: Predict posterior variance ∑(θ) using Equation (14) 4: Compute evaluation value $V[J_{p_{i}}(\theta )P(\theta )]$ using Equation (20) 5: End for 6: Select the $\overset{-}{\theta }$ having the largest evaluation value 7: Return $\overset{-}{\theta }$


Given a likelihood of the uncertain parameters, this regression model is used to inexpensively predict the scores over the total regions of the parameter space, including the sampled set and the not-sampled set. These predictions are combined with the probability distribution of the parameters to estimate the expected reward score. And the samples, obtained through active learning, induce the greatest improvement in GPR model. 

The active learning algorithm can improve the accuracy of the model, but the obvious deficiency is that the iterative process increases the computational cost. However, the purpose of Gaussian process regression and active learning algorithm is to obtain an approximate mapping relation between uncertain parameters and task reward score. As long as the mapping relationship is obtained, it can be used directly in follow-up evaluation process. Next section proposed an improved selection strategy to reduce calculation amount of active learning algorithm.

## 4. Improved AL Algorithm

In order to reduce the computational complexity of AL algorithm, multi-points simultaneous sampling, which selects a batch samples in every iteration, can be carried out to improve the iterative speed of AL algorithm. The disadvantage of multi-points simultaneous sampling is that it may lead to these selected samples being concentrated in some areas of the whole sample space. The information entropy of these samples is relatively high, which means these are the most informative samples, but the existence of sample information redundancy increases the extra sampling cost, from the perspective of training GPR model. Therefore, it is necessary to research an improved mechanism for multi-points simultaneous sampling. This section describes the improved sampling-based algorithm using manifold learning.

### 4.1. Manifold Learning

Zhou [30] embedded the manifold learning algorithm into the AL algorithm to solve classification problems and achieved a better classification effect than the original AL. This is because the improved framework not only takes into account the information entropy criterion of samples, but also considers the distribution of samples [28]. Similarly, we use manifold learning to improve our robust task allocation method. Before using the information entropy evaluation criteria to select the samples, the Manifold-Preserving Graph Reduction (MPGR) is used to select the representative samples. This method can overcome the shortcomings of multi-points simultaneous sampling in the AL algorithm and it can reduce the number of samples without reducing the accuracy of the evaluation, and quickly evaluate the impact of uncertain parameters on the task reward score.

MPGR is a simple and effective graph reduction algorithm based on manifold hypothesis. The manifold hypothesis means that the examples in a small local neighborhood have similar properties. A manifold-preserving sparse graph can be constructed based on MPGR algorithm. This sparse graph could represent the distribution of all samples with a small number of samples, and it can be seen as a discrete representation of the original manifold. A graph with manifold preserving properties means that points outside the sparse graph have highly spatial connectivity with those points in the sparse graph.

According to [29], given a graph G consisting of all unlabeled samples, the manifold-preserving sparse graphs are those that are highly spatially connected to the original graph G. The definition of spatial connectivity is given by Equation (22):

(22) $$\frac{1}{p-s}\sum_{i=1}^{p}\max_{j=1,\ldots ,s}W_{ij}$$

where, p represents the number of all samples, s represents the number of samples retained, W representative weight matrix.

By using McDiarmid inequality, the sparse graph is guaranteed to have high spatial connectivity to a certain extent. The MPGR algorithm approximately maximizes Equation (22) by using the degree. The degree is defined as:

(23) $$d(p)=\sum_{p-q}W_{pq}$$

where, p−q means point *p* and point *q* are connected (whether the two points are connected is according to the K nearest neighbor principle, K-NN), $W_{pq}$ is their corresponding weights defined as Equation (24):

(24) $$W_{pq}=\left\{ \begin{array}{l} \exp(-\frac{||x_{p}-x_{q}|{|}^{2}}{t\eta }),\;\mathrm{if}\;x_{p},x_{q}\;\mathrm{is connected}\\ 0,\;\mathrm{if}\;x_{p},x_{q}\;\mathrm{is not connected} \end{array}\right.$$

where, t is an adjustable parameter, η is the average of the nearest distances (the minimum distance between a point and all its neighbors) of all points. If two points are not connected, their weight is considered to be 0. Due to the simple validity of degree, it is used in the MPGR algorithm as a guide for constructing sparse graphs. The greater the degree of a point, the more information the point has, the more likely it is to be selected to construct a sparse graph.

MPGR is shown as Algorithm 2. First, a graph is constructed from not-sampled set *U* according to K-NN principle (line 2). Then, the degree of each node is calculated in the graph (line 4), and according to the degree-priority principle, a lot of samples are orderly selected (line 5) to make up sparse subset Ls (line 6), which can represent origin *U*. When a certain sample is selected, this sample and corresponding edge in graph are removed from the graph (line 7). The algorithm terminates until enough samples are selected to construct a sparse subset Ls.

**Algorithm 2** Manifold Learning: MPGR1: Input: *U*(*m_t_*); Output: Ls(*m_l_*) 2: Using K-NN construct a graph G from all unlabeled sample points 3: For *i* = 1: *m_l_* do 4: Compute degree d(j), $j=1,\ldots ,m_{t}-i+1$ 5: Select sample $j^{*}=\underset{j\in G}{\arg\max}d(j)$ 6: Ls → add sample $j^{*}$ 7: G → remove sample $j^{*}$ and corresponding side 8: End for 9: Return Ls(*m_l_*)

### 4.2. Improved Sampling Strategy

When the AL multi-points simultaneous sampling method is used to select samples, the information entropy of the selected samples may be very large, but the representation of the selected samples is not strong. These selected samples may be located in some same blocks of whole sample space. And from the perspective of training and learning, there is sample information redundancy. In order to get the regression model better and faster, we can use manifold learning to further improve the selection of training samples.

The main work of the improvement is to use the MPGR method to generate a sparse subset with good representativeness and high degree of spatial connectivity with the original not-sampled set U. Then, the information entropy of this sparse subset is evaluated, and multiple samples with the highest entropy are selected simultaneously. The selected training set has better representation of the set U and higher information entropy. These samples can be used to train GPR model better to acquire a predicted map relationship more approaching to the real one.

The improved AL is shown as Algorithm 3. The sample set is broken up into two subsets. Firstly, a small number of samples are selected from whole sample space and their task reward scores are calculated. Then they are added to the initial training set *S*, and the remaining samples will be regard as not-sampled set U. Then, the algorithm will initially train the GPR model through *S* (line 1), and select samples to train the GPR model iteratively. During each iteration, the MPGR algorithm is used to generate a representative sparse subset Ls (line 4) from U, and then Ns samples with the highest information entropy are selected from Ls to enter *S* for training the GPR model (lines 5–9). Finally, task reward scores of all samples are predicted (line 11), and then the approximate expected reward is calculated using the expectation method (line 12). The approximate expected reward can be used to evaluate the influence of uncertain parameter on task reward scores.

**Algorithm 3** Compute-Expected-Score: Improved AL 1: Input: *U*, *S*, T, N; Output: $\hat{J_{p_{i}}}$ 2: Train a regression model $GP(m(\theta ),k(\theta ,\theta^{\prime }))$ using *S* 3: for each iteration t = 1:T do 4: Call MPGR make Ls from *U* 5: Select best Ns samples according Equation (20) → sampling set $\theta_{s}$ 6: Obtain true scores of $\theta_{s}$ 7: *S*
→ add $\theta_{s}$ 8: *U*
→ remove $\theta_{s}$ 9: Retrain GP model using *S* 10: End for 11: Compute estimated scores ${J^{\hat{k}}}_{p_{i}}=\mu (\theta_{k})$ for all $\theta_{k}\in S\cup U$ 12: Estimate expected score $\hat{J_{p_{i}}}\approx \sum_{k=1}^{N}\omega_{k}\mu (\theta_{k})$ 13: Return $\hat{J_{p_{i}}}$


## 5. Computational Experiments

Simulation experiments was conducted on the hybrid sampling strategy approximations algorithm (active learning and manifold learning). The numerical computations were carried out in Qt5.9.1 on a 2.8GHz, 16GB RAM laptop (Lenovo ThinkPad X1, Beijing, China).

### 5.1. Robust CBBA Simulation

#### 5.1.1. Simulation Setup

The simulation scenario is a multi-UAV formation to perform rescue tasks. Task allocation parameter θ includes UAV location, UAV speed, UAV fuel consumption, mission location, mission value, mission execution time, mission effective time window, etc. There are five UAVs performing rescue tasks with time-window constraints on 10 targets, and the UAVs do not change their flying height during this process, and maintain a constant speed. The time window of each task remains constant. A fully-connected network is adopted for the communication topology.

Robust CBBA is the task allocation method based on CBBA using Monte-Carlo sampling method to calculate expected reward under parameter uncertainty. Deterministic CBBA is to deal with the uncertainty of the allocation parameters based on basic CBBA. That is, the uncertain allocation parameters of the algorithm are replaced by the mean of their distribution. Robust CBBA simulation assumes task duration $t_{jduration}$ obey uniform U(5, 25) distribution while deterministic CBBA uses the average of the distribution parameters ($t_{jduration}$ is 15 s) to run the task allocation algorithm.

The deterministic CBBA and the robust CBBA algorithm are respectively run under the same environment configuration, and the reward calculated from the task assignment method is compared with the reward obtained from the execution of the corresponding allocation results. The average of the total task reward scores using 1000 Monte Carlo simulations for each number of total tasks is used as the final statistical result.

#### 5.1.2. Results and Analysis

**Figure 1:**
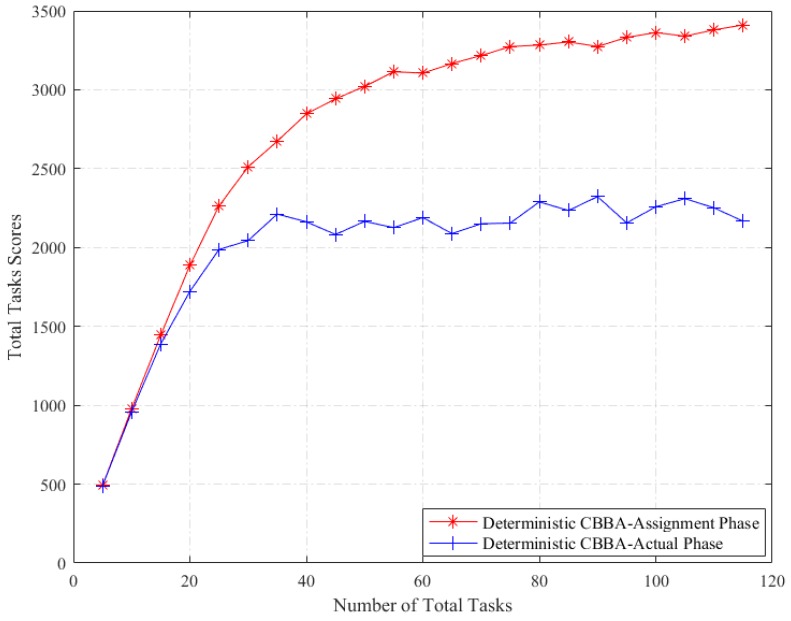
Total tasks scores of Deterministic CBBA: Assignment vs. Execution.

**Figure 2:**
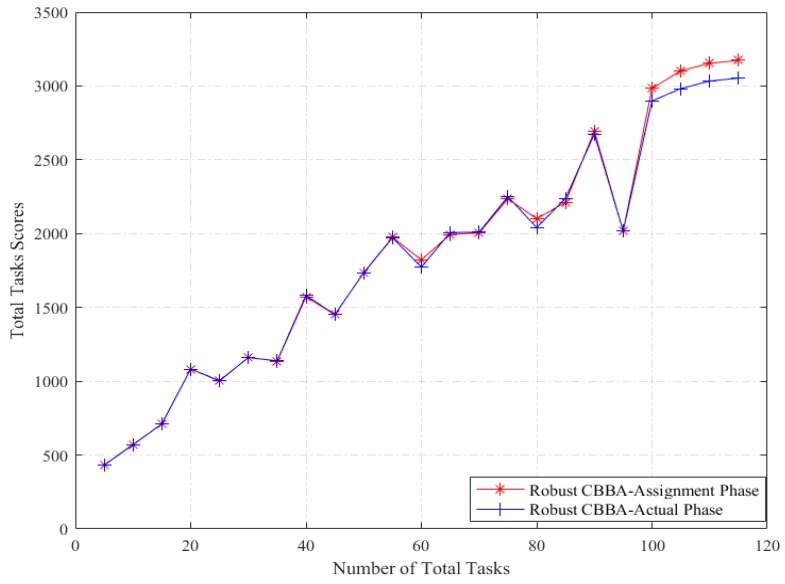
Total tasks scores of Robust CBBA: Assignment vs. Execution.

From Figure 1 and Figure 2, we can see the effectiveness and feasibility of robust CBBA. On the one hand, the deterministic CBBA achieves a higher overall reward under assignment phase than the actual execution phase, while the robust CBBA achieves a similar overall reward under assignment phase and the actual execution phase. On the other hand, in the actual execution phase, robust CBBA achieves a higher reward score than deterministic CBBA. It is due to the robust strategy captures the influence of the task duration uncertainty on the task reward. However, the deterministic CBBA using the mean value of the uncertain allocation parameter fails to capture this uncertainty, and as a result the total scores of the deterministic CBBA have been greatly reduced in the actual execution phase. 

### 5.2. Improved Sampling Strategy Simulations

**Figure 3:**
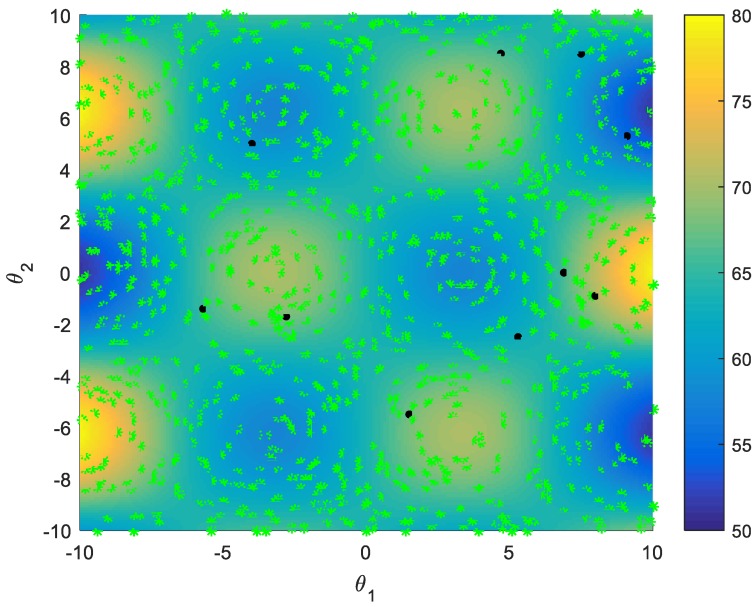
$\theta -J_{p_{i}}(\theta )$ Real mapping relationship (sample set).

The task duration is assumed as an uncertain parameter. When an UAV bids for a new task in the task bundle construction phase, the mapping relationship between the uncertain duration of the newly added task and the task bundle reward score after joining the new task is studied. Different sampling strategies are used to compare the training effects of the GPR model that is used to predict the mapping relationship. In Figure 3, the score corresponds to the color of the area, and green * represent the not-sampled set, and black • represent the initial training set.

**Figure 4:**
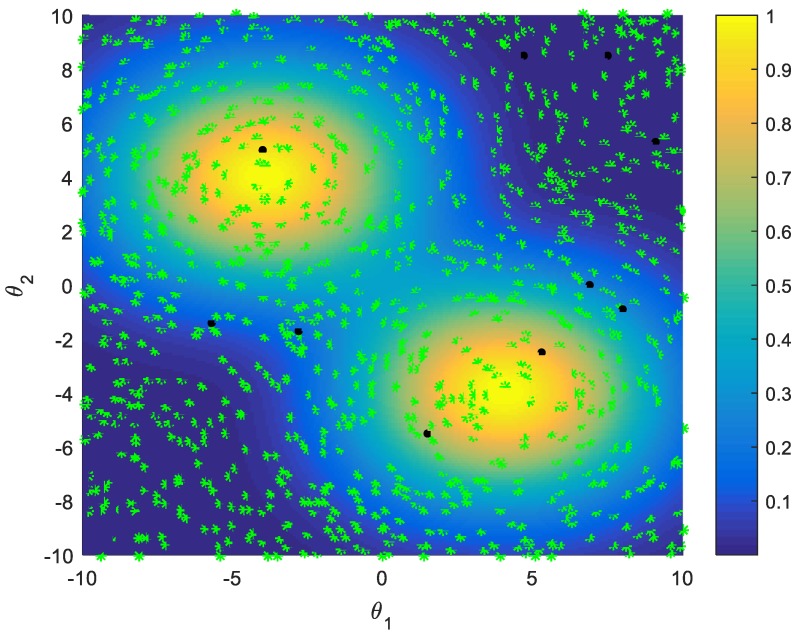
$\theta_{1}$ and $\theta_{2}$ Probability distribution (sample set).

These sampling strategies include random sampling, active learning single-point and multi-points sampling, and improved multi-points sampling strategy. Assume that the task duration is effected by the two parameters $\theta_{1}$ and $\theta_{2}$, and the real relationship between the two parameters and the task score is shown as Figure 3. These two parameters probability distribution is shown as Figure 4. The initial training set of the GPR model consists of 10 samples selected randomly. 

In Figure 4, the probability corresponds to the color of the area. In order to test the effectiveness of the proposed sampling strategy for training the GPR model, the accuracy of the GPR model is mainly observed by two indicators: Relative Error as Equation (25), Relative Root Mean Square Error as Equation (26):

(25) $$RelativeError=|\hat{J_{p_{i}}}-J_{p_{i}}|/J_{p_{i}}$$

where, $\hat{J_{p_{i}}}$ is the expected score calculated approximately based on the prediction of GPR model, $J_{p_{i}}$ is the expected score calculated on 10,000 samples using the Monte-Carlo sampling method.

(26) $$RelativeRMSE=\sqrt{\sum_{k=1}^{N}\omega_{k}{\left(\hat{{J_{p_{i}}}^{k}}-{J_{p_{i}}}^{k}\right)}^{2}}/J_{p_{i}}$$

where, $\omega_{k}$ is the probability weight of sample $\theta_{k}$, $\hat{{J_{p_{i}}}^{k}}$ is the predicted task reward score of sample $\theta_{k}$ based on GPR model, ${J_{p_{i}}}^{k}$ is the true task reward score of sample $\theta_{k}$.

#### 5.2.1. Random Selection Strategy vs. Active Learning Selection Strategy

**Figure 5:**
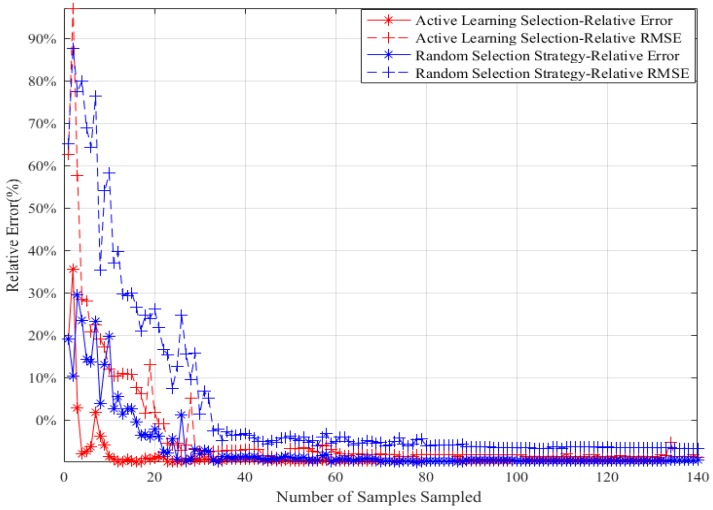
Random Selection Strategy and Active Learning Selection Strategy Simulation Results.

The random selection strategy and the AL selection strategy are used to get a sample to join the training set to train GPR model (single-point sampling) during each iteration. Figure 5 is a relative errors comparison between the random selection strategy and the AL selection strategy. The lateral axis indicates the number of samples selected for joining the training set by each selection strategy, and the vertical axis indicates the two errors of the GPR model. The simulation results show that, for the same number of individuals in training set in iterative process, the active learning selection strategy can finally achieve smaller error than the random selection strategy.

#### 5.2.2. Improved Sampling Strategy vs. Active Learning Strategy on Multi-Points Simultaneous Sampling 

However, for the same number of individuals in the training set in an iterative process, the active learning selection strategy is computationally more burdensome than the random selection strategy. This is due to the fact that an active learning selection strategy selects those samples with the highest information entropy to join the training set, which requires more computation than the random selection strategy. In order to reduce the computational complexity of the entire GPR model training process, a multi-points simultaneous sampling strategy can be considered. The experiment sets multi-points simultaneous sampling to select 10 samples to join the training set during each iteration.

**Figure 6:**
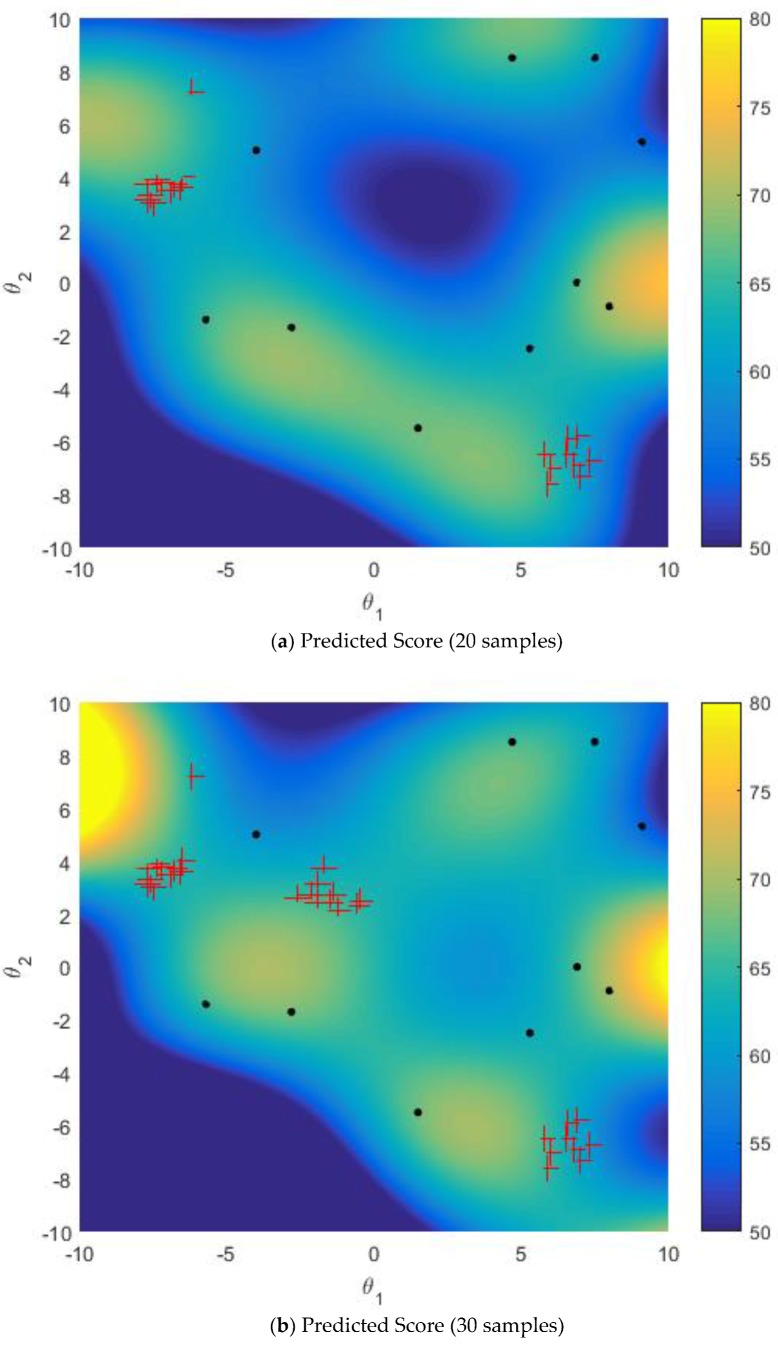
AL multi-points sampling.

**Figure 7:**
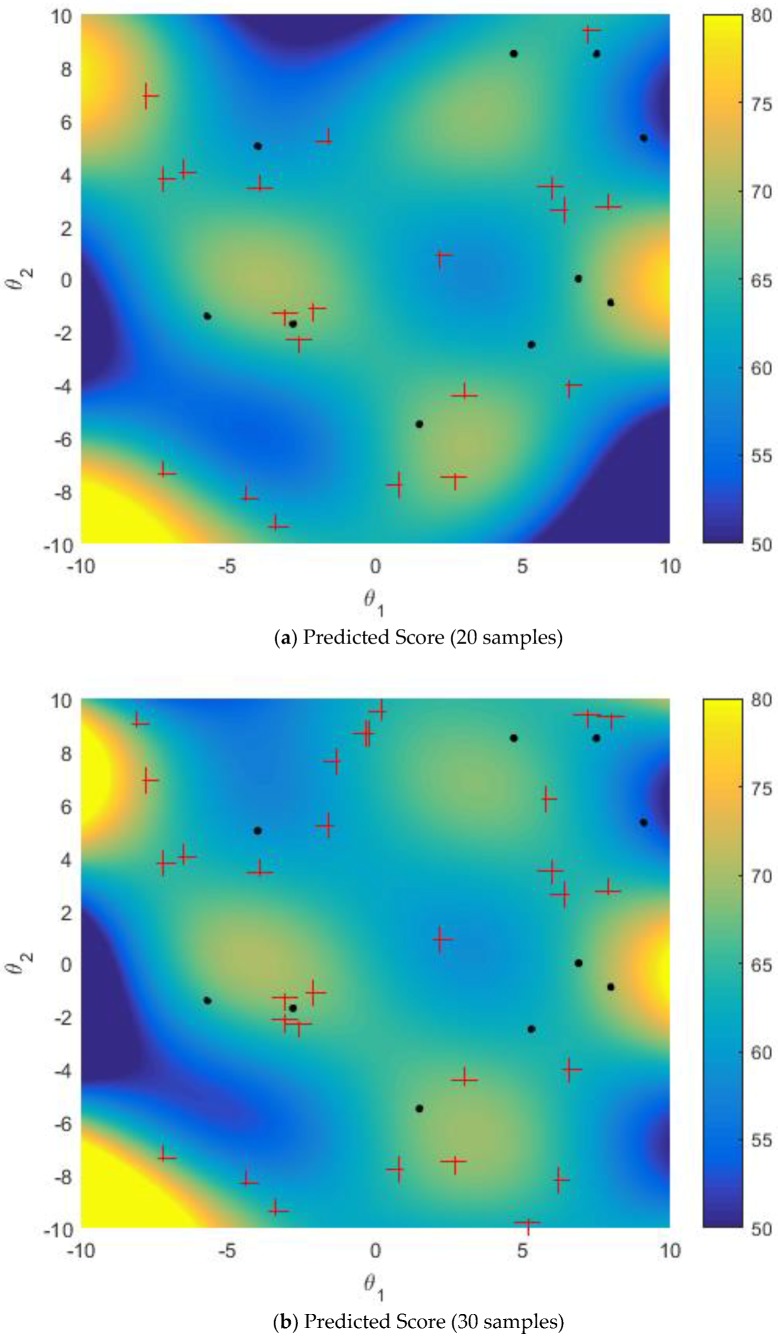
Improved AL Multi-points Sampling.

Figure 6 and Figure 7 show the predicted mapping relationship between uncertain parameters and task reward score when multi-points simultaneous sampling is used in the GPR model training iterative process. For the active learning sampling strategy and the improved sampling strategy, the initial training set includes 10 samples. The simulation results of active learning sampling strategy and the improved sampling strategy are shown in Figure 6 and Figure 7, respectively, where red + represent the selected samples. MPGR algorithm of the improved sampling strategy is used to construct the K-nearest neighbor graph, and the size of sparse subset Ls is 100. In this simulation, each sample point selects the nearest 10 (K = 10) points to form the edge.

When active learning sampling strategy is used for multi-points simultaneous sampling, there will be an agglomeration of samples, shown as Figure 6. Compared with Figure 6, due to the use of the improved sampling strategy, the spatial distribution of the selected sample in Figure 7 is relatively uniform, with no agglomeration, and the overall prediction effect is better. That is to say, the prediction mapping relationship trained by the improved sampling strategy is closer to the true mapping than the single AL sampling strategy. 

**Figure 8:**
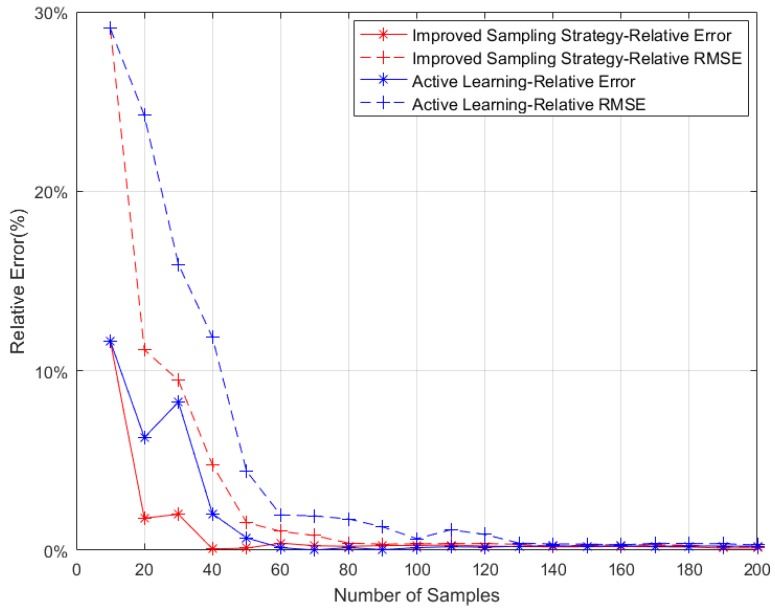
Relative errors of Improved Sampling Strategy and Active Learning Multi-points Simultaneous Sampling Strategy.

Figure 8 is a relative errors comparison between the improved sampling strategy and the AL sampling strategy when multi-point simultaneous sampling is used. The lateral axis indicates the number of samples selected for joining the training set by each selection strategy, and the vertical axis indicates the two errors of the GPR model. 

The simulation results show that, for the same number of individuals in the training set in the iterative process, the improved sampling strategy can finally achieve smaller errors than the AL sampling strategy. That is, compared with AL sampling strategy, the proposed sample selection method can improve the accuracy of the trained model. On the other hand, it also can be seen that, the relative error of improved sampling strategy is close to 0 after 40 samples added into the training set while the AL sampling strategy requires 60 sample added into the training set. And the relative RMSE of improved sampling strategy tends to be stable after 80 samples added into the training set, while the AL sampling strategy requires 130 sample. 

When multiple points are simultaneously sampled to achieve sufficient evaluation accuracy, using the improved sampling strategy to train a GPR model requires a smaller number of samples. In other words, in order to achieve enough accuracy, the improved sampling strategy needs fewer samples to train the GPR model.

#### 5.2.3. Effect of Size of Sparse Subset

**Figure 9:**
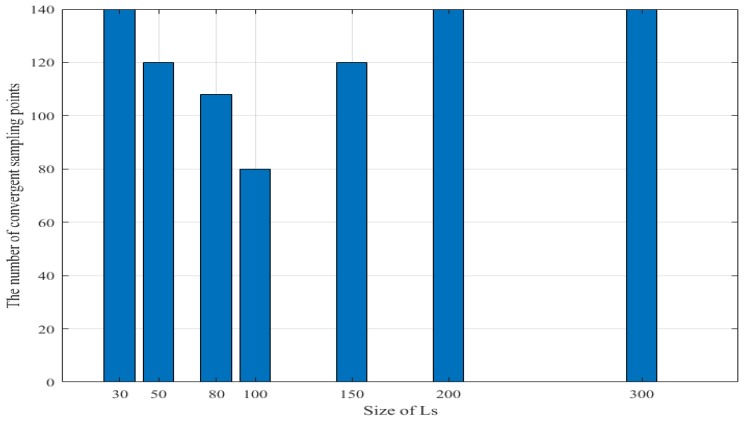
Improved AL Convergence Sampling Quantity.

However, the convergence sampling number (both relative error and relative RMSE are stable) of the improved sampling strategy is related to the size of the subset Ls generated by the MPGR algorithm. If the size of Ls is too small, the selected representative samples carry insufficient information; and if the size of Ls is too large, the selected representative samples have redundant information. Figure 9 shows the relationship between the number of converged sampling points and the size of Ls. For the problem in this paper, it can be seen that the best size of Ls is about 100.

#### 5.2.4. The Comparisons of the Calculation Costs

This paper mainly analyzes three sampling strategies for constructing the training set: active learning single-point sampling, active learning multi-points sampling, and improved sampling strategy multi-points sampling. The significance of the research sample selection method is to reduce the computational load required for training without reducing the accuracy of the GPR model training. So it is necessary to analyze the computational cost of three sample selection methods for training GPR model.

The process of training GPR model is mainly divided into sampling phase and training phase. The calculation cost of the sampling phase is the cost of sample selection of the training set, which need to evaluate the information entropy of the not-sampled set. The calculation cost of training phase is the cost of reward scores calculation, which need to simulate the task reward scores of samples in training set. Additionally, for the improved sampling strategy, there is the filter cost of representative samples. When the number of sample set is large, the main cost is the information entropy evaluation cost and the model training cost. It’s due to the filter of representative samples is based on the degree index of the sparse subset generated by the MPGR algorithm, the K-nearest neighbor graph can be constructed only once, so the correlation calculation cost could be ignored.

Therefore, the calculation cost of these three sampling strategies are compared. The size of initial training set is 10, and the size of not-sampled set is 1000. Table 1 shows the calculation cost of these three sampling strategies when the GPR model is trained to the same relative RMSE (0.2%). Table 2 shows the calculation cost of these three sampling strategies when the same number of samples are selected to construct the training set to train the GPR model. The calculation cost of training phase is represented as number of training, and the calculation cost of sampling phase is represented as number of information entropy evaluation. The final training set includes the initial training set and the samples selected during iterations.

As shown in Table 1, to train GPR model to achieve same relative RMSE, the improved multi-points sampling strategy requires minimum number of total samples of training set. And the number of training is reduced due to the reduction in the number of iterations. Since multi-points simultaneous sampling is used and the information entropy is evaluated only on the sparse subset generated by manifold learning, the number of information entropy evaluation is greatly reduced compared with the other two strategies.

As shown in Table 2, when the number of total samples of training set keeps same for these three sampling strategies, the improved multi-points sampling strategy can achieve smaller relative RMSE, which means a better prediction accuracy of GPR model is trained. At the same time, the associated computational cost is relatively small.

Therefore, if the information entropy calculation cost or the cost of training GPR model is high when the influence of uncertain parameters on the task reward score is evaluated, the improved multi-points simultaneous sampling strategy proposed in this paper can be used to reduce the computational complexity of the algorithm while ensuring the accuracy of the model.

## 6. Conclusions and Further Work

This paper proposed an improved sampling strategy for dealing with the uncertainty of allocation parameters in multi-UAVs task allocation problems. Based on the robust CBBA algorithm and Monte Carlo expectation method, Gaussian process regression and active learning algorithm are used to evaluate sampling-based approximate robustness. Multi-points simultaneous sampling with active learning can reduce the number of training iterations, but it has the disadvantage of agglomeration of samples. To resolve this problem, a preliminary screening strategy using manifold learning methods is used to effectively reduce the computational complexity and improve the accuracy of the algorithm. The simulation results verify the rationality and effectiveness of the proposed improvement strategy.

The improvement strategy proposed in this paper is mainly to combine the screening strategies and multi-points sampling for the training of the Gaussian process regression model serially. This screening method avoids the sample redundancy caused by using a single information entropy selection strategy. The next study may combine the representative selection strategy and the information entropy selection strategy in parallel. The parallel hybrid can reduce the computational complexity more effectively, and enhance the real-time performance of the task assignment algorithm in the case of parameter uncertainty. This is our future work.

## Figures and Tables

**Table 1:** Comparison of sampling methods (achieving the same relative root mean square error).

Sampling Method	Relative RMSE (%)	Number of Iterations	Number of Total Samples of Training Set	Number of Training	Number of Information Entropy Evaluation
active learning single-point sampling	0.20	112	122	$\sum_{i=0}^{112}\left(10+i\right)$	$\sum_{i=0}^{119}\left(1000-i\right)$
active learning multi-points sampling	0.20	13	140	$\sum_{i=0}^{13}\left(10+10i\right)$	$\sum_{i=0}^{12}\left(1000-10i\right)$
improved sampling strategy multi-points sampling	0.20	8	90	$\sum_{i=0}^{8}\left(10+10i\right)$	800

**Table 2:** Comparison of sampling methods (the same number of samples sampled).

Sampling Method	Relative RMSE (%)	Number of Iterations	Number of Total Samples of Training Set	Number of Training	Number of Information Entropy Evaluation
active learning single-point sampling	0.37	80	90	$\sum_{i=0}^{80}\left(10+i\right)$	$\sum_{i=0}^{79}\left(1000-i\right)$
active learning multi-points sampling	0.86	8	90	$\sum_{i=0}^{8}\left(10+10i\right)$	$\sum_{i=0}^{7}\left(1000-10i\right)$
improved sampling strategy multi-points sampling	0.20	8	90	$\sum_{i=0}^{8}\left(10+10i\right)$	800
